# Intercellular adhesion molecule 1 and selectin l play crucial roles in ulcerative colitis

**DOI:** 10.1097/MD.0000000000036552

**Published:** 2023-12-08

**Authors:** Jie He, Zhijie Ni, Zhongbo Li

**Affiliations:** a Department of Colorectal Surgery, China Aerospace Science & Industry Corporation 731 Hospital, Beijing, China.

**Keywords:** bioinformatics, ICAM1, SELL, therapeutic target, ulcerative colitis

## Abstract

Ulcerative colitis (UC) is a chronic inflammatory bowel disease that primarily affects the mucosal layer of the colon (large intestine). However, the relationship between Intercellular Adhesion Molecule-1 (ICAM1), SELL and UC is unclear. The UC datasets, GSE87466 and GSE36807, were downloaded from the gene expression omnibus database. The R package limma was utilized to identify differentially expressed genes (DEGs). Weighted gene co-expression network analysis was conducted. The construction and analysis of protein–protein interaction network, functional enrichment analysis, gene set enrichment analysis, and comparative toxicogenomics database analysis were performed. TargetScan was employed to screen miRNAs regulating central DEGs. Western blot (WB) was used to verify. A total of 2118 DEGs were identified in our study. Gene ontology analysis indicated their enrichment primarily in immune system processes, cellular responses to chemical stimuli, responses to organic substances, responses to external stimuli, and immune responses. Kyoto Encyclopedia of Genes and Genomes analysis revealed that the target cells were mainly enriched in chemokine signaling pathways and TNF signaling pathways. Gene set enrichment analysis enrichment analysis showed significant enrichment in chemokine signaling pathways and cell adhesion molecules. In the Metascape enrichment project, gene ontology terms included regulation of cell activation and positive regulation of immune response. Through the construction and analysis of a protein–protein interaction network, we identified 11 core genes (ICAM1, SELL, CD44, CD40, CCR7, CXCL8, CD19, CCL4, CD274, IL7R, IL1B). We found that the core genes (ICAM1, SELL) were highly expressed in UC samples and lowly expressed in normal samples, suggesting their potential regulatory roles in UC. These core genes were associated with lymphoproliferative disorders, inflammation and necrosis. WB results confirmed the high expression of ICAM1 and SELL in UC. ICAM1 and SELL are highly expressed in UC, and the higher the ICAM1 and SELL genes, the worse the prognosis.

## 1. Introduction

Ulcerative colitis (UC) is a chronic inflammatory intestinal disease that mainly occurs in the mucosal layer of the colon and rectum. It is a type of inflammatory bowel disease (IBD), similar to Krohn disease, but UC only affects the colon and rectum, while Krohn disease can affect any part of the intestine. UC is a common IBD. It typically first appears between the ages of 20 and 40. The incidence rate of UC is similar for men and women and is primarily observed in high-income countries and regions such as North America, Europe, and Australia, especially in northern and eastern Europe. Environmental factors such as smoking, diet, infection, and stress may all be associated with the risk of UC.^[1–4]^ UC is characterized by chronic inflammation of the mucosal layer of the colon and rectum. It does not affect the small intestine and stomach. The inflammation in UC is chronic, continuous, and diffuse. Inflammation can affect the entire colon and rectum or be limited to certain areas. UC can cause symptoms such as diarrhea, abdominal pain, bloody stools, anemia, weight loss, and other.^[5–8]^The initial lesion in UC is inflammation of the colon mucosa, primarily characterized by edema, congestion, and infiltration of inflammatory cells in the mucosal layer. The lesions of UC can also affect the muscular layer of the colon. Prolonged inflammatory reactions can cause degeneration, atrophy, and fibrosis of the intestinal wall. Some patients may experience dysplasia.^[9,10]^ UC is a chronic, recurrent IBD that can lead to intestinal complications, anal complications, malnutrition, psychological impact, and an increased risk of cancer risk for patients. The exact cause of UC is unclear, and this disease may be related to genetic factors, chromosomal abnormalities, gene fusion, and other factors. Therefore, it is particularly important to conduct in-depth research on the molecular mechanisms of UC.

Intercellular Adhesion Molecule-1 (ICAM1) is located on chromosome 19p13.3 to 13.2 and possesses a relative molecular weight ranging from 80,000 to 100,000. It is now recognized as CD54 and is a member of the immunoglobulin superfamily of adhesion molecules, holding a pivotal role in mediating adhesive interactions. ICAM-1 exists in 2 forms: sICAM-1 (soluble) and mICAM-1 (membrane-bound). In its soluble form, sICAM-1 is generated from mICAM-1 through proteolytic cleavage, releasing extracellular components into the bloodstream. Measuring the levels of sICAM-1 in serum can offer insights into the expression status of local ICAM-1. This molecule is primarily participates in leukocyte trafficking and immune cell recruitment. Genes associated with ICAM-1 have gene ontology (GO) annotations related to signal receptor activity and protein complex binding.^[11,12]^ Selectin l (SELL), also known as CD62L, is a cell adhesion molecule belonging to the family of adhesion molecules that mediate the initial interactions between white blood cells (leukocytes) and the endothelial cells lining blood vessels.^[13]^ Due to cell type-specific glycosylation, the actual molecular weight of mature l-selectin can vary between different cell types, ranging from 65 kDa on lymphocytes to 100 kDa on neutrophils, for example. l-Selectin is predominantly expressed on the surface of white blood cells, including lymphocytes and neutrophils, and it plays a crucial role in facilitating the migration of these cells from the bloodstream to inflamed tissues.^[14]^

The connection between ICAM1, SELL genes, and UC is presently shrouded in uncertainty. Hence, this article seeks to employ bioinformatics technology to delve into the central genes distinguishing UC from normal tissues. We will carry out enrichment and pathway analyses to validate the substantial roles of ICAM1 and SELL genes in the context of UC, utilizing a publicly available dataset.

## 2. Methods

### 2.1. UC dataset

Gene expression omnibus (GEO) is a bioinformatics database designed for the storage and sharing of gene expression data and other related biological information. Within the GEO database, retrieval operations are carried out to identify datasets that align with the research interest. These datasets were screened using keywords, biological characteristics, and disease status. The relevant datasets were found and evaluated to ensure compliance with the objectives of the study. Appropriate data sets were selected and data were acquired for subsequent analysis.

In this study, configuration files for UC datasets, GSE87466 and GSE36807, were generated from the GEO database (http://www.ncbi.nlm.nih.gov/geo/)of GPL13158 and GPL570 downloaded from. GSE87466 includes 87 samples of UC and 21 normal samples. GSE36807 includes 15 samples of UC and 7 normal samples. Differentially expressed genes (DEGs) used to identify UC.

### 2.2. Debatch processing

For the merging and de batching of multiple datasets, we initially utilize the R software package to combine the datasets GSE87466 and GSE36807. To merge these datasets effectively, we employ the R software package inSilicoMerging [DOD: 10.1186/1471-2105-13-335] to create a merged matrix. Furthermore, we use the removeBatchEffect function of the R software package limma (version 3.42.2,) to remove batch effects and ultimately obtain the matrix after removing batch effects, which is applied to subsequent analysis.

### 2.3. Screening of DEGs

The R package “limma” is used for probe aggregation and background correction of the merging matrix of GSE87466 and GSE36807. The Benjamin Hochberg method is used to adjust the original *P* value. The false discovery rate (FDR) was used to calculate the fold change. The cutoff value of DEG was *P* < .05 and fold change > 1.5. A visual representation of the volcano map was created.

### 2.4. Weighted gene co-expression network analysis (WGCNA)

Firstly, we employed the debatched merged matrices from GSE87466 and GSE36807 to calculate the median absolute deviation for each gene. Subsequently, we removed the 50% of genes with the smallest median absolute deviation values. We also used the R software package WGCNA goodSamplesGenes method to remove outliers and samples, and further constructed a scale free co expression network using WGCNA. To categorize genes with similar expression profiles into gene modules, we conducted an average linkage hierarchical clustering analysis utilizing the dissimilarity measurement method known as topological overlap matrix. The gene tree graph had a minimum size of 30, and the sensitivity was set to 3. To delve deeper into module analysis, we computed differences in module feature genes, selected a threshold for the module tree view, and merged some modules. In addition, we also merged modules with distances <0.25 and ultimately obtained 20 co expression modules. It is worth noting that the Grey module is considered a gene set that cannot be assigned to any module.

### 2.5. Construction and analysis of protein–protein interaction (PPI) network

Search Tool for the Retrieval of Interacting Genes (STRING) database collect, score, and integrate all publicly available sources of PPI information, and supplement these sources by calculating predictions. In this study, a series of differentially expressed genes were input into the STRING database and constructed a PPI network for predicting core genes (confidence level > 0.4). Cytoscape software can provide biologists with biological network analysis and 2-dimensional visualization. This study visualized and predicted core genes in the PPI network formed by the STRING database using Cytoscape software. Initially, we imported the PPI network into the Cytoscape software and employed MCODE to identify the modules with the most connections. We also used 2 algorithms (MCC and MNC) to calculate the top genes with the most connections and took their intersections. After visualization, we exported the list of core genes.

### 2.6. Functional enrichment analysis

GO analysis and Kyoto Encyclopedia of Genes and Genomes (KEGG) analysis are databases for gene function annotations. They are evaluate tools for assessing gene functions and biological pathways. Input the list of differentially expressed genes selected from the Wayne plot into the KEGG API (https://www.kegg.jp/kegg/rest/keggapi.html). We acquired the latest KEGG Pathway gene annotation to serve as a background and mapped the genes to this background set. For enrichment analysis, we utilized the R software package clusterProfiler (version 3.14.3) for enrichment analysis to obtain the results of gene set enrichment. We also used the GO annotation of genes in the R software package org.Hs.eg.db (version 3.1.0) as a background to map the genes to the background set, with a minimum gene set of 5 and a maximum gene set of 5000. *P* < .05 and FDR < 0.25 are considered statistically significant measures.

In addition, the Metascape database can provide comprehensive gene list annotations and analysis resources, and visually export them. We conducted functional enrichment analysis using the Metascape (http://metascape.org/gp/index.html) database and exported the list of differentially expressed genes mentioned above.

### 2.7. Gene set enrichment analysis (GSEA)

From GSEA (DOI: 10.1073/pnas. 0506580102, http://software.broadinstitute.org/gsea/index.jsp) website for GSEA software (version 3.0), the sample according to UC and normal tissue was divided into 2 groups. Considering gene expression profiles and phenotypic grouping, we set the minimum gene set was set to 5 and the maximum gene set was set to 5000. We conducted a thousand resamplings, and statistical significance was defined as *P* < .05 and FDR < 0.25.

### 2.8. Gene expression heatmap

We utilized the R packet heatmap to generate heatmaps displaying the expression levels of core genes identified by 2 algorithms within the PPI network from GSE87466 and GSE36807. These heatmaps allowed us to visualize the differences in expression of core genes between UC and normal tissue samples.

### 2.9. Comparative toxicogenomics database (CTD) analysis

The CTD database consolidates extensive data on chemical substances, genes, functional phenotypes, and disease interactions, providing significant convenience for investigating disease-related environmental exposure factors and potential drug mechanisms. We input the core gene into the CTD website, found the disease most related to the core gene, and used Excel to draw a radar chart map of the expression difference of each gene.

### 2.10. WB experimental

WB is an experimental technique used to detect the presence and amount of specific proteins in protein mixtures.

### 2.11. Sample handling

Collect samples and lyse cells or tissue samples using protein extraction buffer. Include protease inhibitors in the extraction buffer to prevent protein degradation. After centrifuge of the cell or tissue samples, collect the supernatant to obtain protein extracts.

### 2.12. Protein loading and electrophoresis

Mix the extracted protein mixture with protein loading buffer to ensure uniform protein loading. Then, load the protein samples into the wells of an SDS-PAGE gel and conduct electrophoresis to separate proteins on their size.

### 2.13. Protein transfer

Transfer the separated proteins from the SDS-PAGE gel to a protein transfer membrane, typically using a semi-dry transfer method. Utilize transfer buffer for the transfer, often at low temperature.

### 2.14. Membrane handling

Rinse the transfer membrane in TBST to eliminate any residual SDS and protein loading. Block nonspecific binding sites, usually with 5% nonfat milk or 3% BSA in TBST.

### 2.15. Antibody reaction

Incubate the membrane with the primary antibody to recognize the target protein. Wash the membrane to remove unbound primary antibody. Subsequently, incubate the membrane with the secondary antibody, which is typically enzyme- or fluorescence-labeled. Wash the membrane once more to eliminate any unbound secondary antibody.

### 2.16. Visualization and imaging

Utilize a substrate for visualization to observe protein bands on the membrane. The selection of substrate depends on the labeling of the secondary antibody. Expose X-ray film or employ a digital imaging system to capture protein bands on the membrane.

### 2.17. Analysis and quantification

Utilize molecular weight protein standards to ascertain the molecular weight of the target protein. Quantify the relative protein content of the target protein using image analysis software or an imaging system.

### 2.18. miRNA

TargetScan is an online database for prediction and analyzing miRNAs and their target genes. In our study, TargetScan was used to screen miRNAs that regulate central DEG. Analyzing miRNA (microRNA) provides a profound understanding of the roles and functions of small RNA molecules. miRNAs represent a class of non-coding RNA molecules that play pivotal roles in gene regulation, cellular signaling, disease mechanisms, and more.

## 3. Result

### 3.1. Screening of DEGs

**Figure 1.**
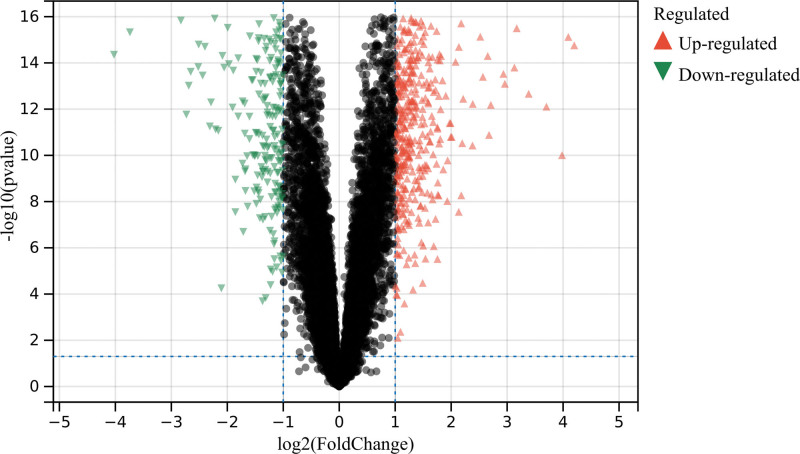
Analysis of differentially expressed genes. (A) A total of 2118 DEGs.

In this study, a total of 2118 DEGs were identified based on the de batch combination matrix of GSE87466 and GSE36807 (Fig. 1).

### 3.2. Functional enrichment analysis

#### 3.2.1. Functional enrichment analysis of DEGs.

**Figure 2.**
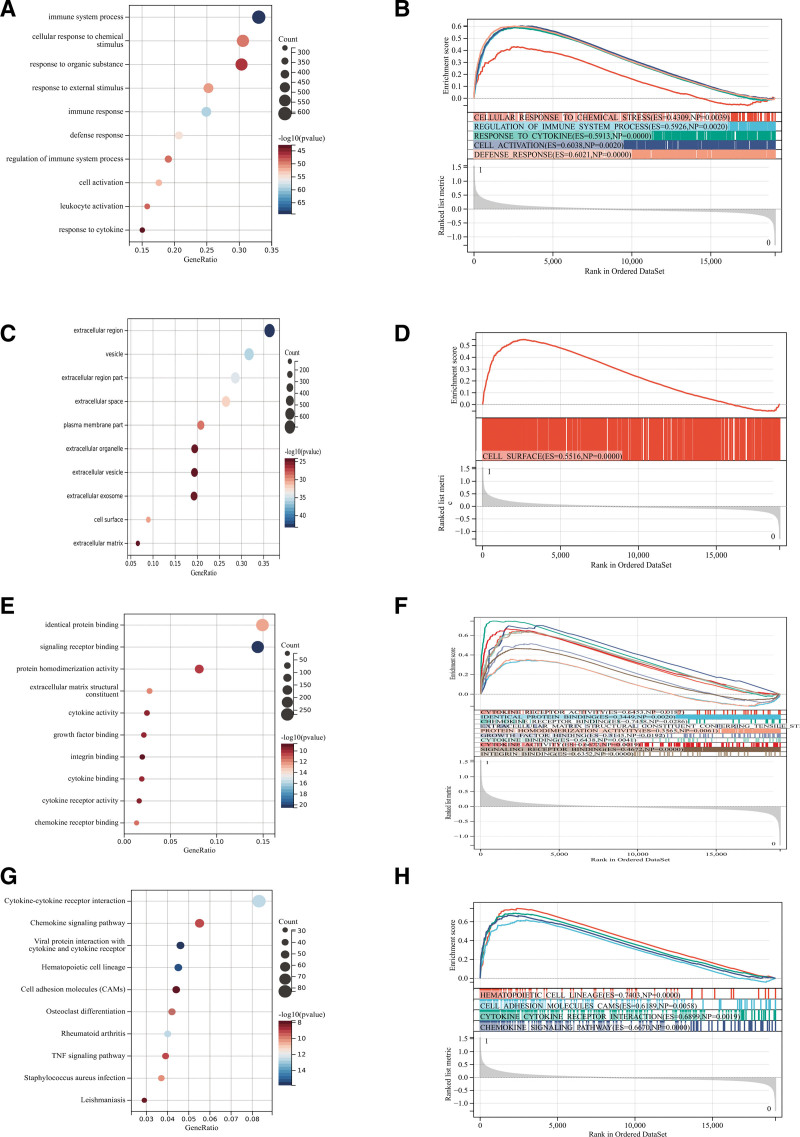
Functional enrichment analysis. (A) GOBP analysis (C) GOCC analysis (E) GOMF analysis (G) KEGG analysis (B, D, F, H) GSEA enrichment analysis, and the intersection diagram of enrichment items and GO and KEGG enrichment items of differentially expressed genes. GO = gene ontology, GSEA = gene set enrichment analysis. KEGG = Kyoto Encyclopedia of Genes and Genomes.

We conducted GO and KEGG analyses on these differentially expressed genes. According to GOBP analysis, they were primarily enriched in immune system processes, cell responses to chemical stimuli, responses to organic substances, responses to external stimuli, and immune responses (Fig. 2A). According to GOCC analysis, they were mainly enriched in extracellular regions, cysts, extracellular regions, extracellular spaces, and plasma membrane regions (Fig. 2C). According to GOMF analysis, they were mainly enriched in protein binding, signal receptor binding, protein homodimerization activity, extracellular matrix structural components, and cytokine activity (Fig. 2E). According to KEGG analysis, these target genes were mainly enriched in the chemokine signaling pathway and TNF signaling pathway (Fig. 2G).

#### 3.2.2. Gene set enrichment analysis.

In addition, we performed GSEA enrichment analysis on the entire genome with the goal of identifying potential enrichment items in non-differentially expressed genes and validating the results of differentially expressed genes. The intersection of enrichment terms and GO KEGG enrichment terms of the differentially expressed genes depicted in the figure, primarily enriched in the chemokine signal pathway and cell adhesion molecule (Fig. 2B, D, F, H).

#### 3.2.3. Metascape enrichment analysis.

**Figure 3.**
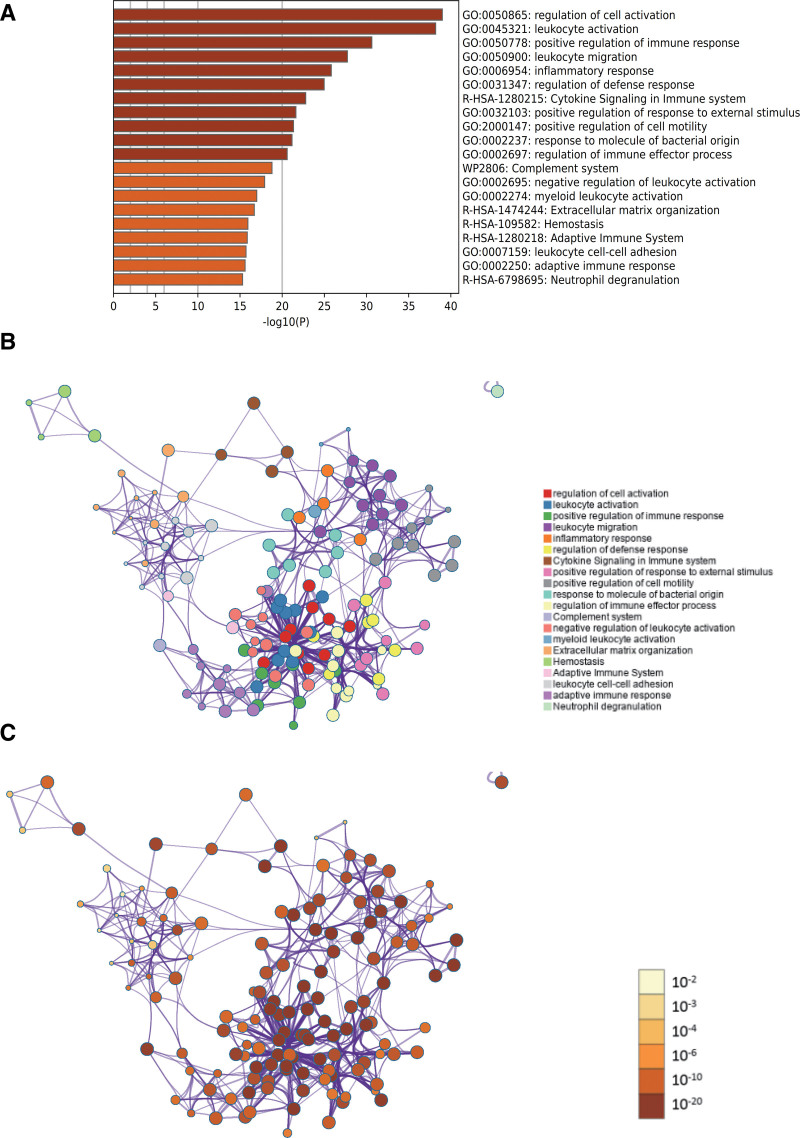
Metascape enrichment analysis. (A) GO enrichment item, GO has the regulation of cell activation, the active regulation of immune response (B and C)The enrichment network colored by enrichment term; the enrichment network colored by *P* value. GO = gene ontology.

The enriched content of Metascape includes GO enriched items (Fig. 3A), in which GO regulates cell activation and actively regulates immune responses. Simultaneously, we generated an enrichment network color-coded with enrichment terms and *P* values (Fig. 3B and C) to visualize the correlations and confidence levels of each enrichment item.

#### 3.2.4. Weighted gene co-expression network analysis.

**Figure 4.**
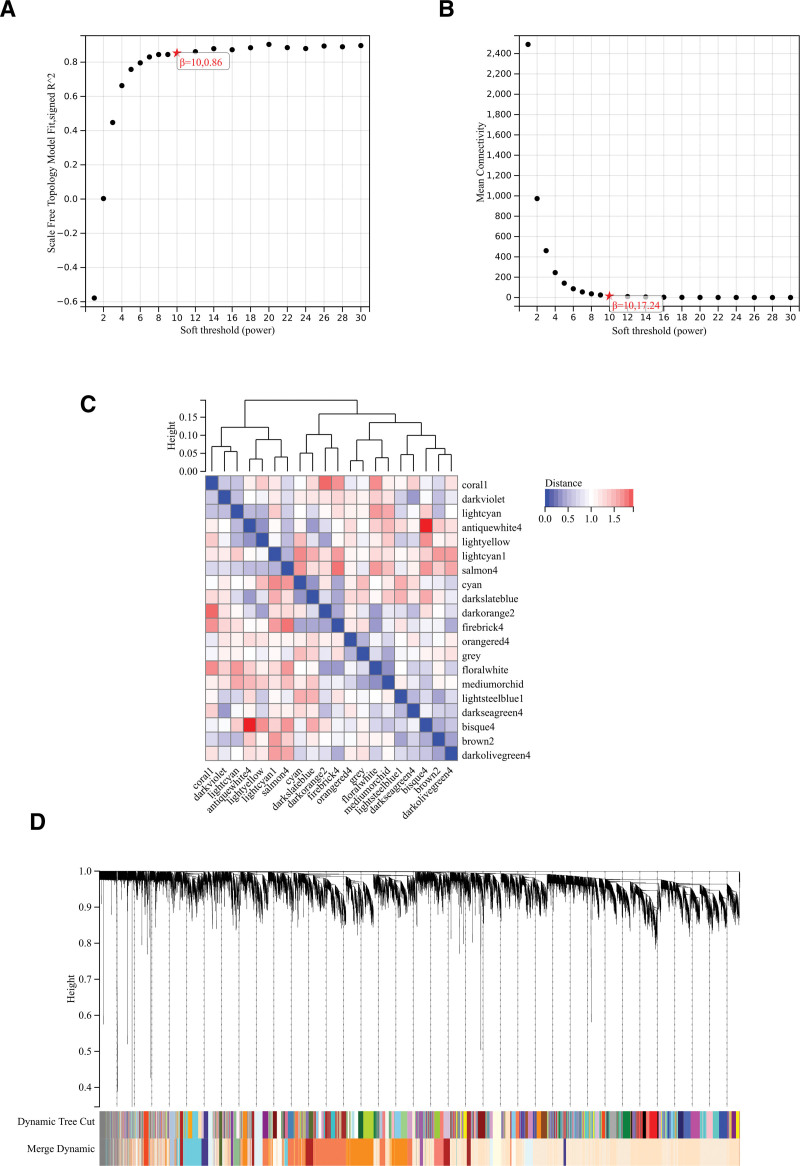
WGCNA. (A) β = 10,0.86. (B) β = 10,17.24 (C) interactions between important modules (D) construct a hierarchical clustering tree of all genes and generate important modules. WGCNA = weighted gene co-expression network analysis.

**Figure 5.**
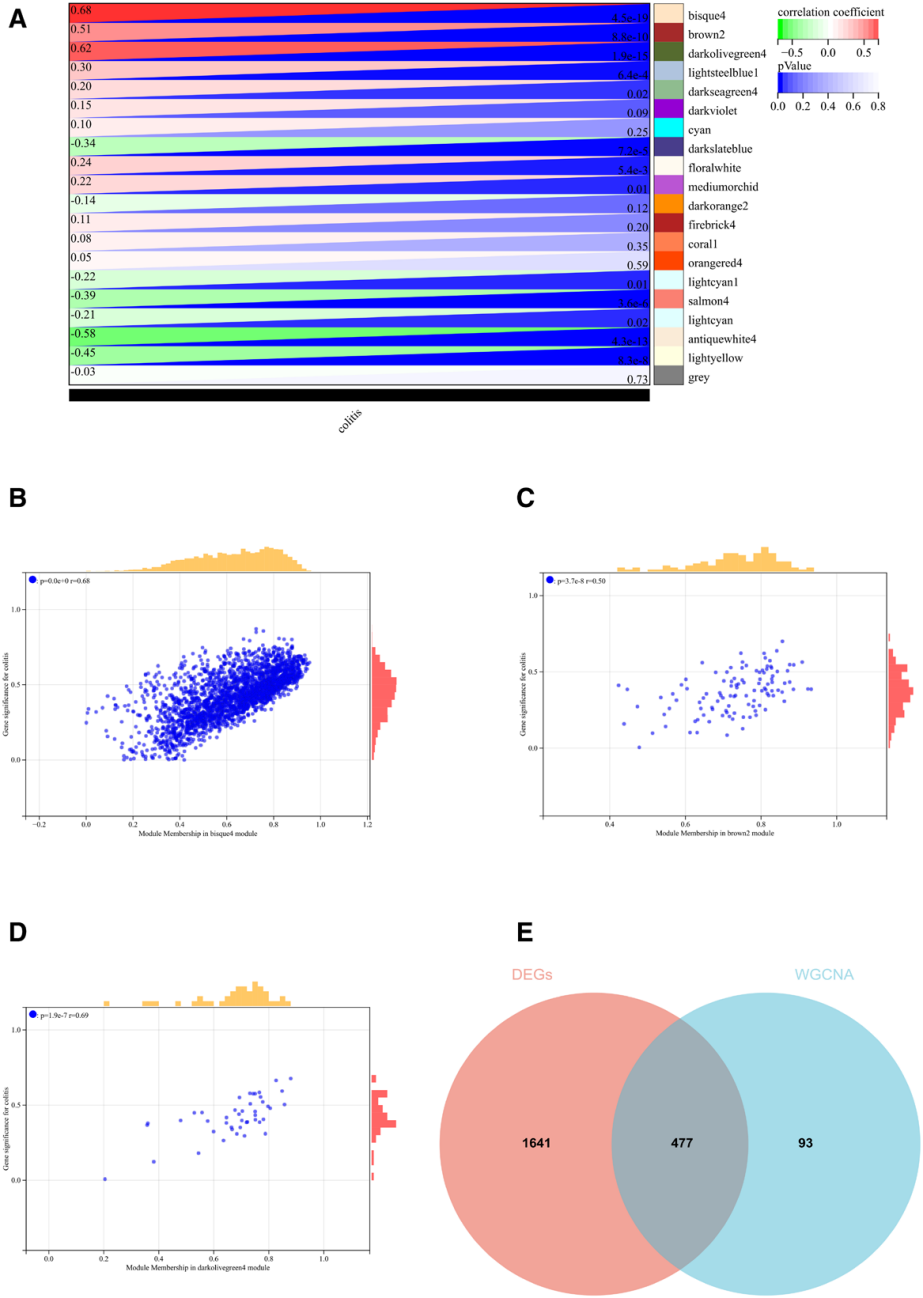
WGCNA. (A) The module phenotypic correlation heat map (B) GS-MM correlation scatter map of related hub genes: P = .0e-0 R = 0.68 (C) GS-MM correlation scatter map of related hub genes: P = 3.7e-8 R = 0.5 (D) GS-MM correlation scatter map of related hub genes: P = 1.9e-7 R = 0.69. GS-MM = Gene Significance-Module Membership, WGCNA = weighted gene co-expression network analysis.

The selection of soft threshold power is a critical step in WGCNA analysis. The soft-thresholding power was determined through network topology analysis. In the WGCNA, the soft-thresholding power was set to 10 (Fig. 4A and B). Hierarchical clustering tree of all genes was constructed, and important modules were generated (Fig. 4D). Then analyzed the interaction between these modules (Fig. 4C). Heat maps of module phenotypic correlations were generated (Fig. 5A).

In WGCNA, Gene Significance (GS) and Module Membership (MM) play a crucial role in identifying and prioritizing genes associated with specific traits or diseases, as well as in characterizing the functional relevance of genes within co-expression modules. These measures assist researchers in discovering potential biomarkers and comprehending the biological mechanisms underpinning complex traits or diseases. GS measures the correlation between the expression of a specific gene and a particular external trait or phenotype, quantifying the gene relevance or significance to the given trait. Genes with high GS for a trait are potentially associated with that trait and warrant further investigation. MM, on the other hand, evaluates the correlation between the expression profile of a gene and the module eigengene, which essentially represents the first principal component of a module. MM indicates how well a gene aligns with a specific module. Genes with high MM in a module are considered core or representative genes within that module. A scatter plot of GS and MM correlation for related hub genes (Figs. 5B–D).

We combined modules with distances <0.25, resulting in a total of 20 co-expression modules. We computed the correlation between module feature vectors and gene expression to obtain MM. Following the established cutoff criteria (| MM |>0.8), we identified 571 genes with strong connectivity in clinically significant modules, designating them as central genes.

We also plotted and intersected the differential genes identified by WGCNA and DEGs for the creation and analysis of protein interaction networks (Fig. 5E).

### 3.3. Construction and analysis of PPI network

**Figure 6.**
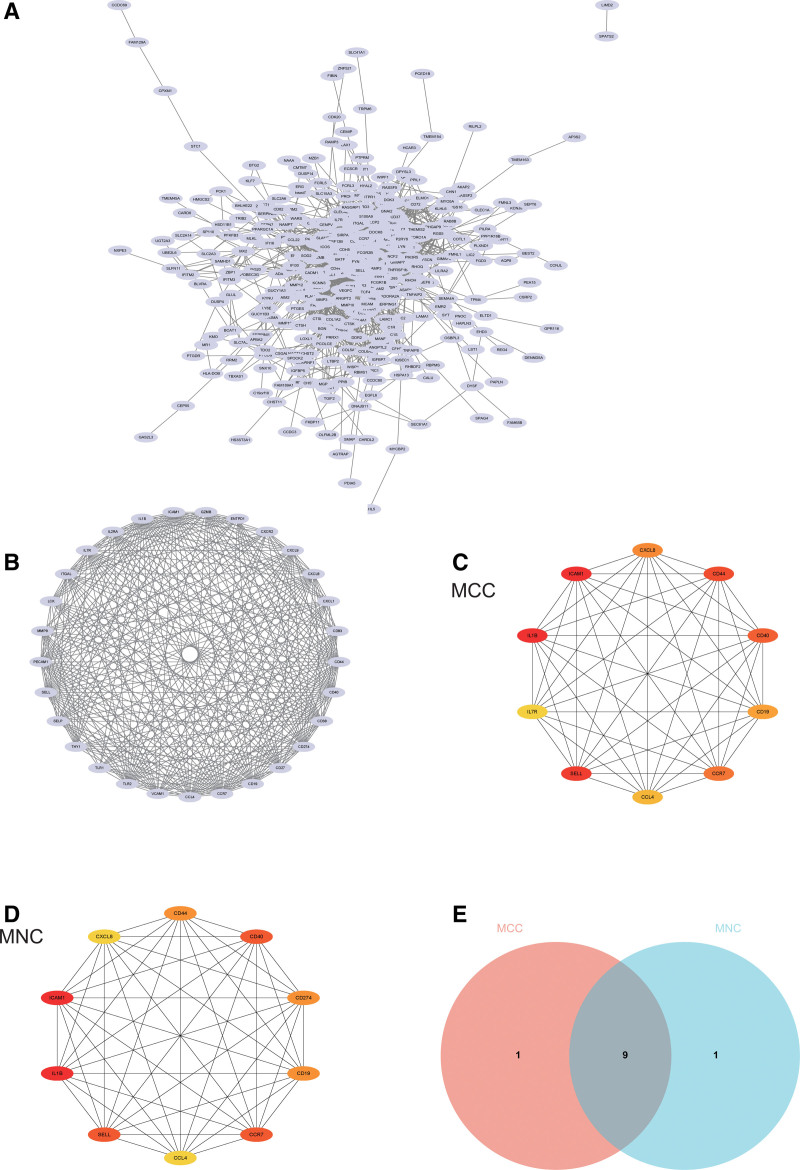
Construction and Analysis of protein–protein interaction (PPI) network. (A) DEGs PPI network was constructed from STRING online database and analyzed by Cytoscape software. (B) Identification central gene (C and D) two algorithms (MCC, MNC) identified central genes (E) Wayne diagram intersection diagram. STRING = Search Tool for the Retrieval of Interacting Genes.

The PPI network of DEGs was constructed using the STRING online database and analyzed with Cytoscape software (Fig. 6A). Core gene clusters (Fig. 6B) were derived. Two different algorithms (MCC, DMNC) were employed to identify the central genes (Fig. 6C and D), and the intersection set was obtained using a Venn diagram (Fig. 6E). This resulted in 11 core genes (ICAM1, Sell, CD44, CD40, CCR7, CXCL8, CD19, CCL4, CD274, IL7R, and IL1B).

### 3.4. Gene expression heatmap

**Figure 7.**
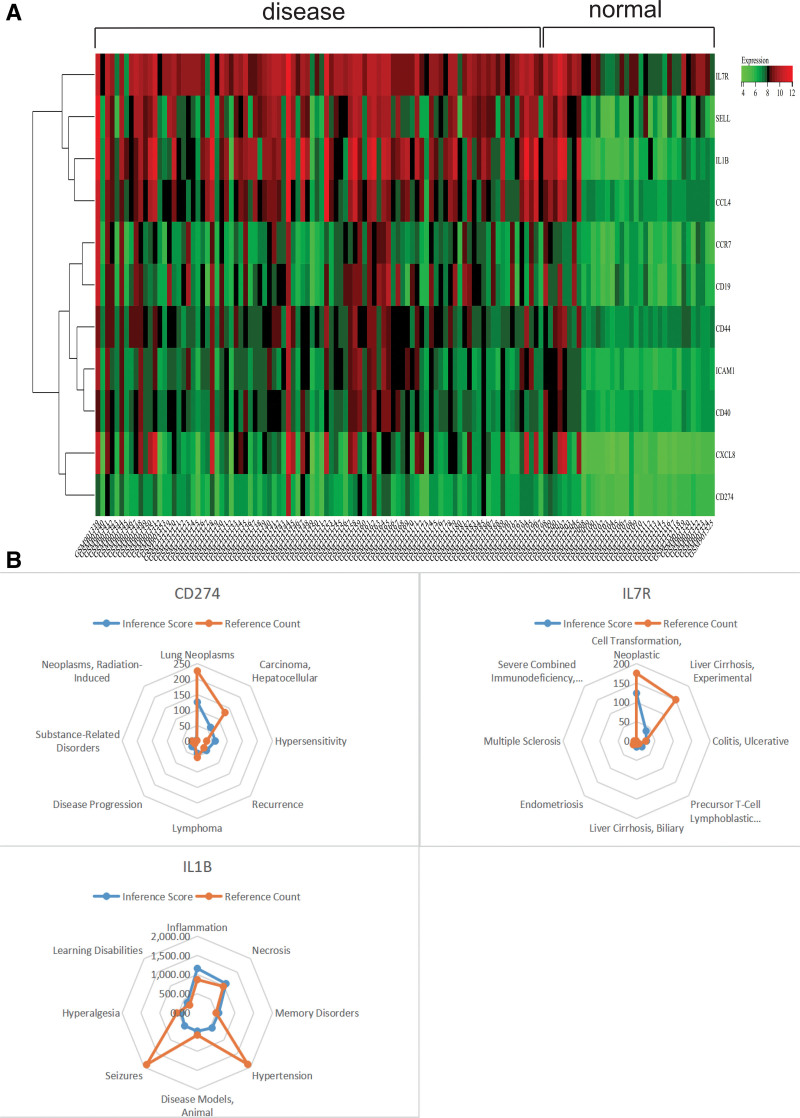
Gene expression heat map. (A) Differential expression of core genes between ulcerative colitis and normal tissue samples (B) CTD analysis, core genes (ICAM1, SELL, CD44, CD40, CCR7, CXCL8, CD19, CCL4, CD274, IL7R, IL1B) are associated with lymphoproliferative diseases, inflammation, lymphoproliferative diseases, and necrosis. CTD = comparative toxicogenomics database, ICAM1 = Intercellular Adhesion Molecule-1.

The differential expression of core genes between UC and normal tissue samples is depicted in the heatmap (Fig. 7A). We observed that 11 core genes (ICAM1, SELL, CD44, CD40, CCR7, CXCL8, CD19, CCL4, CD274, IL7R, IL1B) exhibited high expression levels in UC samples, while they were lowly expressed in normal samples. This suggests that they may have regulatory effects on UC.

### 3.5. CTD analysis

**Figure 8.**
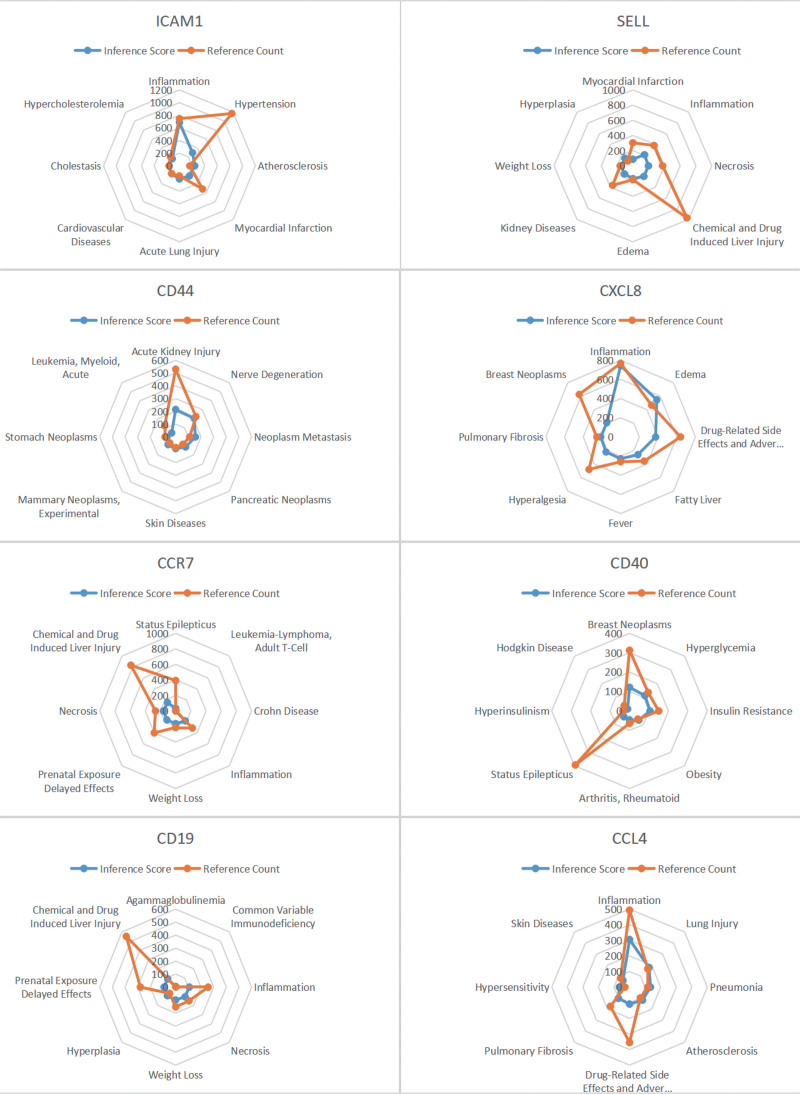
CTD analysis. ICAM1, SELL, CD44, CD40, CCR7, CXCL8, CD19, CCL4 genes were associated with lymphoproliferative diseases, inflammation, lymphoproliferative diseases and necrosis. CTD = comparative toxicogenomics database, ICAM1 = Intercellular Adhesion Molecule-1.

In this study, we inputted a list of hub genes into the CTD website to search for diseases related to core genes and improve the understanding of the association between genes and diseases. Eleven core genes (ICAM1, SELL, CD44, CD40, CCR7, CXCL8, CD19, CCL4, CD274, IL7R, IL1B) were found to be associated with lymphoproliferative diseases, inflammation and necrosis (Figs. 7B and 8).

### 3.6. Prediction and functional annotation of miRNAs related to hub genes

In this study, we input a list of hub genes into TargetScan to search for relevant miRNAs and improve the understanding of gene expression regulation (Table 1). We found that the associated miRNAs of ICAM1 gene are hsa-miR-873-5p. 2 and hsa-miR-377-3p; the miRNAs associated with the CD44 gene are hsa-miR-199a-3p, hsa-miR-199b-3p, and hsa-miR-3129-5p; the associated miRNAs of the CD40 gene are hsa-miR-5195-3p and hsa-miR-145-5p; the miRNAs associated with the CCR7 gene are hsa-miR-4458, hsa-let-7i-5p, and hsa-let-7g-5p; the miRNAs associated with the CCL4 gene is hsa-miR-24-3p; the associated miRNAs of CD274 gene is hsa-miR-140-3p.2; the miRNAs associated with the IL7R gene is hsa-miR-455-3p.1.

**Table 1:** A summary of miRNAs that regulate hub genes.

	Gene	MIRNA		
1	ICAM1	hsa-miR-873-5p.2	hsa-miR-377-3p	
2	SELL	none		
3	CD44	hsa-miR-199a-3p	hsa-miR-199b-3p	hsa-miR-3129-5p
4	CD40	hsa-miR-5195-3p	hsa-miR-145-5p	
5	CCR7	hsa-miR-4458	hsa-let-7i-5p	hsa-let-7g-5p
6	CXCL8	none		
7	CD19	none		
8	CCL4	hsa-miR-24-3p		
9	CD274	hsa-miR-140-3p.2		
10	IL7R	hsa-miR-455-3p.1		
11	IL1B	None		

ICAM1 = Intercellular Adhesion Molecule-1.

### 3.7. WB analysis

**Figure 9.**
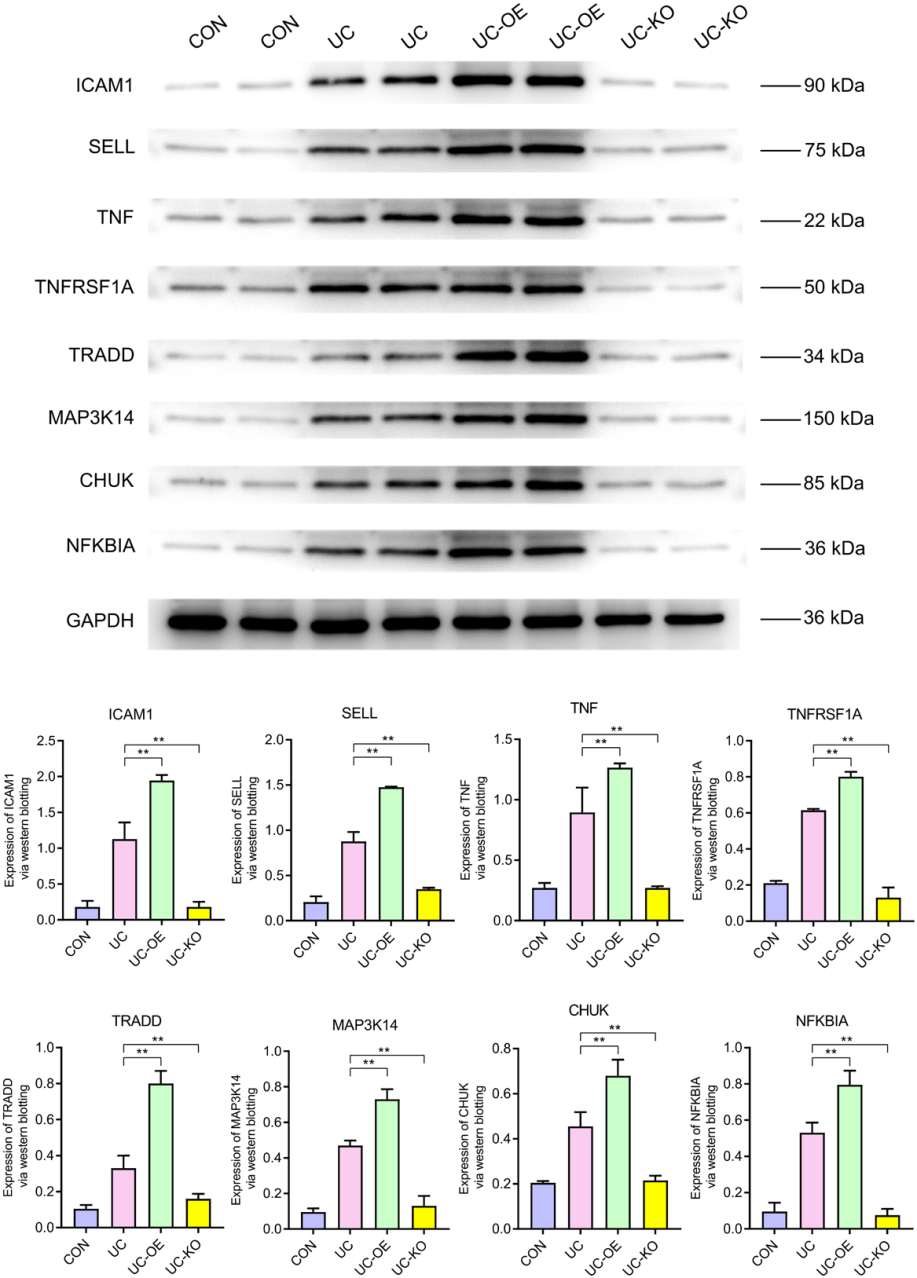
Western blot experiment.

WB results revealed that core genes ICAM1, SELL, TNF, TNFSF1A, TRADD, MAP3K14, CHUK, and NFKBIA exhibited higher expression levels in UC compared to the control group. Additionally, when compared to UC, the UC-overexpression group showed overexpressed, while the UC-knockout group exhibited underexpression (Fig. 9).

## 4. Discussion

UC is a chronic and recurrent IBD that can cause various harms to patients. Chronic inflammatory reactions can lead to complications such as ulcers, stenosis, and perforation of the intestinal mucosa, and in severe cases, can pose a life-threatening risk to patients. UC is often accompanied by anal inflammation, leading to discomfort such as anal pain, difficulties in defecation, and itching of the skin around the anus. Chronic symptoms like diarrhea, constipation, and abdominal pain can affect the patient appetite and their digestive and absorption functions, resulting in issues such as malnutrition and anemia. The chronic and recurrent nature of UC can bring psychological stress and anxiety to patients, affecting their overall quality of life. Patients with UC may also experience atypical hyperplasia and precancerous lesions during long-term inflammatory reactions and intestinal remodeling, which increases their risk of developing colon cancer. UC is an IBD, and its pathogenesis is exceedingly complex, involving multiple molecular mechanisms. Immune system abnormalities in patients with UC, including autoimmune responses, immunosuppressive, and cytokine abnormalities, contribute to the damage of the intestinal mucosa.^[15]^ Dysbiosis of the gut microbiota is a key factor in the pathogenesis of UC, as it leads to leads to the overgrowth of pathogenic microorganisms, compromises the intestinal barrier function, and triggers inflammatory reactions.^[16–19]^ In patients with UC, the integrity of the intestinal epithelial cell barrier is compromised, resulting in erosion and ulceration of the intestinal mucosa. This damage to the epithelial barrier can also allow the infiltration of intestinal endotoxins and the release of inflammatory factors, further exacerbating the inflammatory response.^[20–23]^ Apoptosis plays a crucial role in the pathogenesis of UC, as both inflammatory cells and epithelial cells may undergo apoptosis during the inflammatory response, leading to the destruction and dysfunction of intestinal tissue structure.^[24–27]^ UC may be associated with specific genetic variations, such as NOD2, IL23R, among others. Mutations in these genes can lead to alterations in mechanisms like immune system dysfunction and gut microbiota imbalance, leading to inflammatory reactions and tissue damage.^[28–30]^ A comprehensive exploration of the molecular mechanisms UC is vital for the development of targeted drugs. The primary finding of this study is that the ICAM1 and SELL genes are highly expressed in UC. Elevated expression levels of ICAM1 and SELL genes are associated with a worse prognosis.

ICAM1 is an almost ubiquitous transmembrane glycoprotein, typically expressed at low levels in endothelial cells and immune cells. As a transmembrane protein found in leukocytes and endothelial cells, ICAM1 plays a vital role in stabilizing cell-cell interactions and facilitating the migration of leukocyte and endothelial cells.^[31]^ ICAM-1 is intricately involved in immune cell interactions, including processes such as T cell activation, antigen presentation, and immune synapse formation. Furthermore, it contributes to homeostatic immune responses, ranging from mediating leukocyte trafficking to forming immune synapses and facilitating tissue wound healing.^[32]^ The primary function of ICAM-1 is to mediate cell adhesion, playing a crucial role in promoting adhesion at inflammatory sites and regulating the body immune response. ICAM-1 enhances the adhesion of leukocytes, inflammatory cells, and tumor cells to endothelial cells by specifically binding to its receptor. This, in turn, promotes the activation of endothelial cells and facilitates the penetration of endothelial cells.^[33]^

ICAM-1, belonging to the immunoglobulin superfamily, is a cell surface glycoprotein and adhesion receptor. It plays a pivotal role in regulating the recruitment of white blood cells from the circulation to the inflammatory sites. ICAM-1 is involved in critical physiological processes, including cell signal transduction, activation, immune response, and inflammatory reactions.^[34]^ As a major regulator of various cellular functions, the ICAM-1 promoter contains binding sites for multiple transcription factors. It is engaged in significant signaling pathways that upregulate transcription in response to various stimuli. ICAM-1 functions are essential in processes related to inflammation, injury resolution, and tumorigenesis.^[35]^ ICAM1 acts as a ligand for a variety of integrins, facilitating interactions between immune cells and endothelial cells during inflammation. It is crucial for promoting leukocyte adhesion, migration, and their recruitment at sites of infection or tissue injury.

ICAM-1 plays a crucial role in human defense against infections and the regulation of immune cell trafficking. It is closely associated with various pathological processes, particularly those related to inflammatory response and immune-mediated diseases.^[36]^ Studies have demonstrated that the upregulation of ICAM-1 in endothelial cells, induced by various inflammatory factors during inflammation, enhances leukocyte adhesion and tissue infiltration. This phenomenon is linked to the pathogenesis of diseases such as such as atherosclerosis, rheumatoid arthritis, multiple sclerosis, and IBD. Notably, increased mucosal concentrations of soluble ICAM-1 (sICAM1), sE-selectin, and interleukin-8 have been observed in active UC.^[37]^ Therefore, ICAM-1 plays an important role in UC.^[38]^

SELL (selectin l) is a membrane glycoprotein widely expressed on the cell surface of what in humans and other animals. The expression of SELL is regulated by various factors, including cytokines, viral infections, inflammatory reactions, etc.^[39]^ SELL is primarily expressed on the surface of endothelial cells. During inflammatory and immune responses, SELL can be upregulated, mediating the migration and infiltration of white blood cells.^[40]^ l-Selectin, or SELL, plays a vital role in the initial “rolling” adhesion of white blood cells on the inner surface of blood vessels. This process allows these cells to scan for chemotactic signals and select the appropriate sites for tissue entry. l-Selectin is an integral component of the immune system, enabling immune cells to circulate in the bloodstream while continually surveilling the body for signs of infection or tissue damage. The expression of l-Selectin on the surface of immune cells can be regulated by various factors. Additionally, its interactions with other adhesion molecules such as integrins further promote immune cell recruitment and migration.^[41]^

l-Selectin, a type I transmembrane cell adhesion molecule, t is prevalent on most circulating white blood cells. It is commonly referred to as a tethering/rolling receptor. The binding of l-selectin to endothelial ligands initiates the tethering and rolling behavior of neutrophils on the post-capillary venule walls, marking the first step in a multi-step adhesion cascade. l-Selectin plays a crucial role in facilitating the entry of lymphocytes and neutrophils into sites of inflammation, actively contributing to the body defense mechanisms and tissue repair processes during inflammatory responses.^[42,43]^ Downstream signaling through l-selectin can influence the behavior of neutrophils, affecting adhesion, migration, and activation processes, thereby regulating monocyte protrusion during transendothelial migration. Furthermore, l-selectin and its ligands also participate in the adhesion of the blastocyst to the maternal interface of the uterine endometrium.^[44]^ Relevant studies have demonstrated that SELL expression is significantly increased at the lesion site, correlating with the infiltration and metastasis of inflammatory cells.^[45]^ l-Selectin ligands have been found to impact the progression of colon cancer.^[46]^ Therefore, it is speculated that SELL may play important roles in the inflammation and immune response process of UC.

Although this article has conducted rigorous bioinformatics analysis, there are still some shortcomings. This study did not conduct animal experiments on gene overexpression or knockout to further validate its function. Therefore, in future research, we should conduct in-depth exploration in this area.

## 5. Conclusion

In summary, ICAM1 and SELL are highly expressed in UC and may play a significant role in the development of this condition through cellular regulation and other pathways. ICAM1 and SELL could serve as molecular targets for precise treatment of UC, offering a certain direction for the further research into the mechanisms of this disease. This study has the potential to make a substantial impact on the research and treatment of UC. It contributes to the trend of towards personalized UC treatment, enabling the selection of the most suitable treatment approach based on patients’ disease severity and genotype, thus enhancing treatment effectiveness while reducing side effects. Additionally, it propels the development of new drugs and treatment methods aimed at alleviating UC symptoms and mitigating disease progression. Furthermore, it aids in a better understanding of the disease mechanisms and provides information for early diagnosis and risk assessment.

## Author contributions

**Conceptualization:** Jie He, Zhijie Ni, Zhongbo Li.

**Data curation:** Jie He, Zhijie Ni, Zhongbo Li.

**Formal analysis:** Jie He, Zhijie Ni, Zhongbo Li.

**Funding acquisition:** Jie He, Zhijie Ni, Zhongbo Li.

**Investigation:** Jie He, Zhijie Ni, Zhongbo Li.

**Methodology:** Jie He, Zhijie Ni, Zhongbo Li.

**Project administration:** Jie He, Zhijie Ni, Zhongbo Li.

**Resources:** Jie He, Zhijie Ni, Zhongbo Li.

**Software:** Jie He, Zhijie Ni, Zhongbo Li.

**Supervision:** Jie He, Zhijie Ni, Zhongbo Li.

**Validation:** Jie He, Zhijie Ni, Zhongbo Li.

**Visualization:** Jie He, Zhijie Ni, Zhongbo Li.

**Writing – original draft:** Jie He, Zhijie Ni, Zhongbo Li.

**Writing – review & editing:** Jie He, Zhijie Ni, Zhongbo Li.
